# The Inclusion of the Food Microstructural Influence in Predictive Microbiology: State-of-the-Art

**DOI:** 10.3390/foods10092119

**Published:** 2021-09-08

**Authors:** Davy Verheyen, Jan F. M. Van Impe

**Affiliations:** 1 BioTeC+, Chemical and Biochemical Process Technology and Control, Department of Chemical Engineering, KU Leuven, Gebroeders de Smetstraat 1, 9000 Ghent, Belgium; davy.verheyen@kuleuven.be; 2 OPTEC, Optimization in Engineering Center-of-Excellence, KU Leuven, 3000 Leuven, Belgium; 3 CPMF2, Flemish Cluster Predictive Microbiology in Foods—www.cpmf2.be, 9000 Ghent, Belgium

**Keywords:** predictive microbiology, food microstructure, food safety, mathematical models

## Abstract

Predictive microbiology has steadily evolved into one of the most important tools to assess and control the microbiological safety of food products. Predictive models were traditionally developed based on experiments in liquid laboratory media, meaning that food microstructural effects were not represented in these models. Since food microstructure is known to exert a significant effect on microbial growth and inactivation dynamics, the applicability of predictive models is limited if food microstructure is not taken into account. Over the last 10–20 years, researchers, therefore, developed a variety of models that do include certain food microstructural influences. This review provides an overview of the most notable microstructure-including models which were developed over the years, both for microbial growth and inactivation.

## 1. Introduction

Predictive microbiology encompasses the development of mathematical models to evaluate and predict the effect of environmental conditions (e.g., temperature, pH, CO_2_ level, salt concentration, water activity) on the (growth, survival, and inactivation) behaviour of microorganisms in food (model) systems [1,2]. While some simple model types have been developed over the years, most predictive models are continuous dynamical models that consist of a set of ordinary differential equations (ODEs) [3]. The models are useful tools to assess and design processing, distribution, and storage operations to assure the microbiological safety and quality of food products [1,2]. To date, predictive models have been widely accepted by food producers, governments, and scientists as a sound scientific approach to accomplish legal food safety requirements [4]. The main advantages of predictive microbiology over traditional challenge tests are (i) an increased efficiency regarding financial costs, labour-intensiveness, and time, and (ii) the cumulative nature of the developed models [5]. 

**Figure 1:**
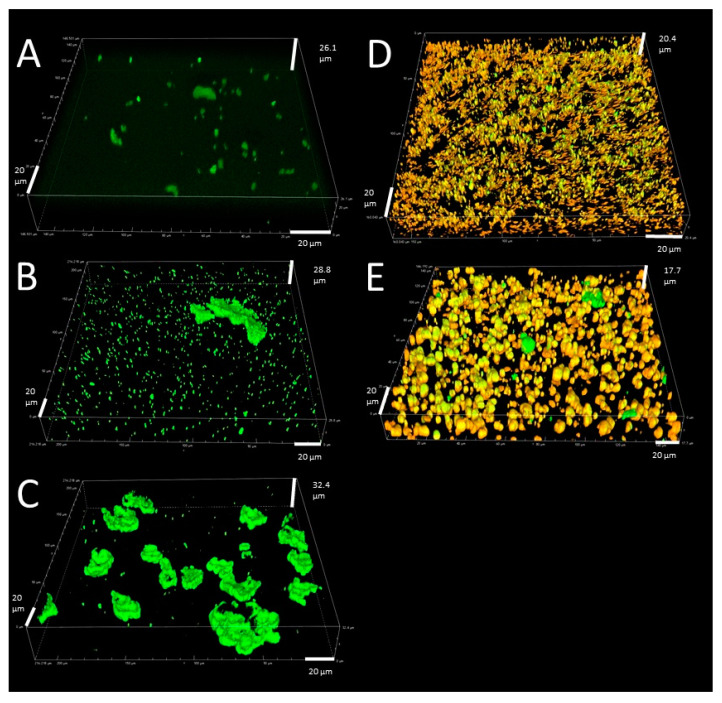
Confocal laser scanning microscopy images of growth of *Listeria monocytogenes* in food model systems with varying microstructural complexity (adapted from [13], with permission from American Society for Microbiology—Journals, 2021). Bacterial cells and fat droplets are shown in green and orange, respectively. The liquid/gelled aqueous phase was not stained and is, hence, shown by the absence of colour. The growth morphology of the bacteria clearly depends on the microstructural aspects of the food (model) system: (**A**) single cells, small aggregates and small microcolonies in a simple low-viscosity liquid system; (**B**) a large number of small aggregates and small microcolonies and some larger microcolonies in a liquid system with increased viscosity; (**C**) microcolonies of different sizes in an aqueous gel model system; (**D**) small aggregates and microcolonies growing in the spaces between fat droplets and around the fat droplets in an emulsion model system; (**E**) small aggregates and microcolonies growing in the spaces between the fat droplets and around the fat droplets in a gelled emulsion model system.

Food structure, from natural or process-generated origins, is defined as the spatial arrangement of the structural elements of food products and their interactions [6,7]. Food structural elements can be interpreted at different scales, i.e., the molecular level (e.g., sugar, water, protein, and polysaccharide molecules), the nanoscale level (e.g., casein micelles), the microscale level (fat and water droplets in emulsions, granules, gel networks), and the macroscale level (e.g., air pockets, powders, foams) [7,8,9]. When investigating microbial behaviour, it is mainly the microscale level (i.e., food microstructure) which is of interest, with influencing aspects including physical constraints on the mobility of microorganisms, variations in oxygen availability, and nutrient diffusion related to the nature of the food matrix (i.e., viscous or gelled, rheological properties), and the presence of fat droplets inside the food matrix [10,11]. An important aspect of the microstructural influence on microbial dynamics is the effect on the growth morphology of microorganisms [12]. Depending on the specific microstructural complexity of foods, microorganisms can occur as single cells, small aggregates (i.e., radius < 1.5 µm), microcolonies (i.e., radius < 200 µm), macrocolonies (i.e., radius > 200 µm), and biofilms [13]. An example of this microstructural influence on bacterial growth morphology is illustrated in Figure 1. 

Traditionally, most predictive models are developed based on experiments in liquid laboratory media, in which case the effect of the food microstructure on the microbial behaviour, albeit a major influencing factor, is not taken into account [10,14]. The applicability of these models is, hence, limited to liquid foods with a relatively uniform distribution of nutrients and microorganisms occurring in a planktonic form [15,16]. In/on more structured foods (e.g., aqueous gels, emulsions, gelled emulsions), model predictions can be either fail-safe (i.e., predicting more growth or less inactivation than in reality) or fail-dangerous (i.e., predicting less growth or more inactivation than in reality) from a food safety point of view [10]. For microbial growth, most liquid-based models are considered fail-safe in/on more structured foods, while for microbial inactivation, most models are considered fail-dangerous [17,18,19,20]. Nevertheless, exceptions to the general trend have been reported for both microbial growth and inactivation in structured foods, meaning that including the food microstructural effect into predictive models would be beneficial for the overall accuracy and safety of predictive models [17,18,21,22]. 

During the 1990s and early 2000s, a few review papers brought to attention the lack of knowledge concerning the effect of the food microstructure on microbial dynamics in the context of predictive microbiology, also proposing modelling frameworks to address the issue in the future [10,23]. During the 20–30 years following on these pioneering works, however, the number of developed predictive models that incorporated food microstructural effects remained scarce. While some useful models have been reported, most of them mainly consisted of isolated efforts more focussed on specific applications (e.g., predicting the growth of microorganisms as a function of the gelatine concentration of the food [24]) rather than systematic modelling frameworks. Nevertheless, a significant amount of useful experimental and modelling-related approaches/concepts have been developed during these last decades. No relevant review papers on this topic have, however, been published in recent years, except for the review of Skandamis and Jeanson [25], focussing on the inclusion of the effect of the type of growth of the microorganisms (i.e., colonial vs. planktonic) into mathematical models for liquid, semi-liquid, and solid foods and food surfaces. Therefore, in the current review, the aim is to provide an extensive overview of the most promising food microstructure-including predictive models which were developed over the years, both for microbial growth and inactivation. For consistency and clarity purposes, the parameters and variables in the different presented models were occasionally renamed to remain uniform among the different examples. 

## 2. Historical Overview on the Inclusion of Food Microstructure in Predictive Models

### 2.1. The Absence of Food Microstructure in the Early Days of Predictive Microbiology

**Figure 2:**
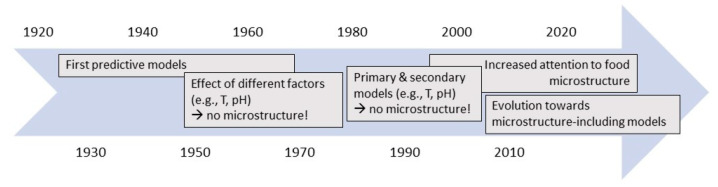
Timeline depicting the evolution on the inclusion of food microstructure into predictive models over the years.

Figure 2 depicts the evolution of the inclusion of food microstructural factors in predictive models over the years. In the early days of predictive microbiology, research did not specifically focus on the influence of food microstructure on microbial behaviour. One of the basic assumptions of predictive microbiology (which is still valid now) was that the growth or inactivation behaviour of microorganisms can be described/predicted based on a limited number of variables. Traditionally, researchers did not see food microstructure as one of those variables and focussed on factors such as temperature, pH, water activity (*a_w_*), nutrient concentration, and the presence of preservatives [26]. Because of this reason, predictive models were mainly developed based on experiments in liquid laboratory media due to their ease of use during microbiological experiments [27].

Predictive growth models, both kinetic and probabilistic, started to appear in the 1960s and 1970s, describing the influence of factors such as storage temperature, salt concentration, and pH [28,29,30]. A renewed interest in predictive models arose in the 1980s, mainly due to a number of major food poisoning outbreaks and the consequent public (and political) awareness of the importance of food safety [1]. This renewed interest culminated in the identification of effective primary models (e.g., modified Gompertz model [31,32], Baranyi and Roberts model [33]), allowing the objective description of growth curves as mathematical equations. From then on, the development of secondary models describing the influence of important environmental factors on the parameters of the primary growth models was possible [26]. Such secondary models were incorporated into primary models to include the effect of those environmental factors on microbial growth into the predictions [34,35,36,37]. Over the years, secondary models became more sophisticated, describing the effect of different factors (e.g., temperature, pH, water activity, acid concentration) and the interaction between them on the growth behaviour of microorganisms [38,39,40]. However, research in the 1990s and early 2000s more and more brought the insight that such models could only adequately predict microbial growth in simple liquid food products. Microbial growth in/on more structured food products generally did not follow the predictions, with both fail-safe and fail-dangerous discrepancies being reported [10,41,42,43]. Extensive research on the effects of food microstructure on microbial growth was conducted in the following years, but models were only scarcely developed [17]. 

The most well-known early predictive model for the inactivation of microorganisms, although originating from before the introduction of the term predictive microbiology, was the *botulinum* cook of Esty and Meyer [44], describing a thermal process designed to kill 10^12^ spores of *Clostridium botulinum* type A [1]. This model was based on the Thermal Death Time (TDT) concept of Bigelow [45], which involves the use of D-values (i.e., the decimal reduction time, or the time necessary to accomplish a reduction in bacterial numbers of one log unit under isothermal conditions) and z-values (i.e., increase in temperature necessary to accomplish a 10-fold decrease in the D-value). Over the years, models based on this concept of D and z-values, such as the *botulinum* cook, have been used extensively in the food industry, with the canning industry being the most notable example [46]. However, the success of this approach in the canning industry is mostly due to overprocessing rather than due to modelling accuracy [47]. The models assume loglinear behaviour according to Equation (1) [48].

(1) $$\log N=\log N_{0}-\frac{t}{D_{T}}$$

with *N*, the cell density at time t; *N*_0_, the initial cell density; *D_T_*, the thermal reduction time (D-value). Similar to predictive growth models, secondary models describing the influence of important factors on the D-value of the primary inactivation model (Equation (1)) were developed. Arrhenius-type models represent one of the earliest methods to develop such secondary inactivation models (e.g., [49,50]). D-values can be expressed by means of the Arrhenius equation, as shown in Equation (2) [51].

(2) $$k_{max}=\frac{2.303}{D_{T}}=A\cdot exp\left(\frac{-E}{R\cdot T}\right)$$

with *k_max_*, the maximum specific inactivation rate; *A*, the frequency factor; *E*, the activation energy; *R*, the gas constant; *T*, the absolute temperature. This general Arrhenius equation can be extended with extra terms to, in addition to the influence of temperature, also include other factors such as pH and *a_w_* [51,52], as for example already applied in the late 1970s by Davey et al. [53] for the effect of temperature and pH during thermal inactivation. The generalised form of such Arrhenius-type secondary models is represented by Equation (3) (based on [51]).

(3) $$\ln k_{max}=a_{0}+a_{1,1}\cdot V_{1}+\cdots +a_{1,n}\cdot V_{1}^{n}+a_{2,1}\cdot V_{2}+\cdots +a_{2,n}\cdot V_{2}^{n}+\cdots +a_{x,1}\cdot V_{x}+\cdots +a_{x,n}\cdot V_{x}^{n}$$

with *n*, the order of the secondary model; *a*_0_–*a_x_*_,*n*_, constants; *V*_1_–*V_x_*, environmental factors such as 1/*T*, *a_w_* or pH. Theoretically, secondary models, be it Arrhenius-type models or other model types, could include food microstructural factors, but this was not the case in the early days of predictive microbiology. Therefore, early models (i.e., including both primary and secondary models) were accurate when describing the inactivation behaviour of microorganisms in simple systems, but were inaccurate in complex food environments because the influence of food microstructure was not taken into account [46]. Again, model predictions could be fail-safe or fail-dangerous, depending on the specific situation [47,54,55]. In order to solve the possible inaccuracy of loglinear predictive inactivation models, new models were developed to deal with common non-loglinear inactivation trends, with the most notable models dating from after 1988 (e.g., [56,57,58,59,60,61,62,63,64]). Some of these models are able to implicitly include the microstructural effect by representing accurate inactivation dynamics in complexly structured food environments, e.g., via shoulder and/or tailing effects. However, a direct modelling of the food microstructural effect on microbial inactivation behaviour is not accomplished by the models if no food microstructural factors are included in the secondary models.

### 2.2. More Attention to Food Microstructure in the Last Decades

Following on the increasing number of studies that showed the significant influence of food microstructure on microbial dynamics, some concepts have been introduced to attempt to develop predictive models which take the food microstructural influence into account.

A relatively straightforward method to include the influence of food microstructure into predictive models is the development of models which are only valid for specific food products. In this case, microbial growth or inactivation experiments are conducted in/on the target food product. This methodology was occasionally already applied in the 1980s and 1990s, but has been widely used in recent literature. An extensive list of example studies exploiting this approach is provided in Table 1, both for microbial growth [65,66,67,68,69,70,71,72,73,74,75,76,77,78,79,80,81,82,83,84,85,86] and thermal inactivation [55,87,88,89,90,91,92,93,94,95,96,97], with a focus on early and recent examples. Interestingly, however, this predictive microbiology approach bears some similarities to the traditional challenge testing approach, in which microbial growth/inactivation experiments were also conducted directly in/on the food product of interest [5]. Hence, this approach could result in a large amount of models only suitable for a specific set of conditions (i.e., food product under certain environmental conditions), as also illustrated by the extensive (yet incomplete) list in Table 1. To the opinion of the authors, this food-specific approach, while certainly valuable, is not completely in line with the initial philosophy of predictive microbiology, which aims for “the accumulation of knowledge on microbial behaviour in foods” [1]. Additionally, and most relevant for this review, while those models inherently take the influence of food microstructure on microbial dynamics into account, they do not describe the influence of food microstructural factors on microbial behaviour [21].

An alternative approach to include the effect of food microstructure into predictive models is conducting experiments in artificial food model systems which simulate, to a certain extent, the microstructure of the product. In general, artificial food model systems are advantageous due to (i) their use being more simple and less labour-intensive than real food products, (ii) the absence of background microflora, (iii) repeatability of experimental results, (vi) the possibility to alter factors independently, and (v) the straightforward transferability of findings to other food products [15,21,98]. Wilson et al. [10] defined six categories of food architectures which could all be represented by artificial food model systems, i.e., liquids, oil-in-water emulsions, water-in-oil emulsions, aqueous gels, gelled emulsions, and surfaces. The use of liquid food model systems (e.g., meat broth to simulate meat products) was already a common practice in the early days of predictive microbiology, mimicking mostly the composition, pH, and water activity of the food products of interest [28,54,99,100,101,102]. In the early 2000s, a large number of research groups underwent a paradigm shift towards the use of more structured model systems. Artificial model systems of different structures are frequently used to study the effect of certain influencing factors on microbial growth [11,15,103,104,105,106,107,108] or inactivation [109,110,111]. Seldomly, predictive models are developed based on experiments in those more structure model systems. In this regard, two approaches can generally be identified, i.e., (A) using one model system to model (or include into the model) the effect of certain environmental or food intrinsic factors (e.g., oxygen diffusion and heat transfer in structured foods) (e.g., [98,112,113,114,115]) and (B) using model systems varying in certain compositional or microstructural factors to include the effect of those factors in relevant secondary models (e.g., [24,27,116,117]). Both approaches A and B can be regarded as systematic approaches to develop predictive models which take food microstructure into account, although not all models developed according to these approaches would explicitly model the microstructural effect. For instance, one could develop a model which describes the influence of the salt content on microbial growth in structured products. This hypothetical model would be valid in structured products, but would not directly express the influence of any microstructural factor. The following sections provide an overview of existing models which include food microstructural effects, for microbial growth and inactivation, respectively.

## 3. Growth Models Incorporating Food Microstructure

To the best knowledge of the authors, all existing growth models which include food microstructural effects can be divided into two categories. The first category encompasses the introduction of macroscale secondary models describing the influence of food microstructural factors on microbial growth, while the second category encompasses semi-mechanistic microscale models which take the local (structured) environment of the bacterial cell(s) into account. Table 2 provides an overview of the most relevant models of the two categories. A more detailed description of the respective models can be found in the following sections.

### 3.1. Macroscale Secondary Models Including Food Microstructural Factors

The traditional approach which has been employed to model the effect of food microstructure on microbial growth dynamics is the development of secondary models based on food microstructural factors of food products. This approach can be regarded as a macroscopic simplification of the real microbial behaviour. In essence, heterogeneity (i.e., both concerning the bacterial distribution and the food environment) is ignored and an average macroscopic relation is assumed. Due to its relative straightforwardness and limited required computing power, this secondary modelling approach has been dominant in predictive microbiology over the years. A remarkable observation is that, in all of the relevant studies focussing on secondary models for the food microstructural influence on bacterial growth, gelatine concentration was used as a variable to include the influence of product rheology on the maximum specific growth rate *µ_max_* in the Baranyi and Roberts growth model [24,27,118].

Antwi et al. [24] developed a predictive model for the growth of *Listeria innocua* and *Lactococcus lactis* (mono- and coculture), quantifying the influence of a gelatine gel matrix. For this purpose, the model of Vereecken et al. [123], describing bacterial growth in function of undissociated lactic acid concentration and pH, was fit to growth data in model systems containing different gelatine concentrations. After their model optimisation procedure, Antwi et al. [24] obtained the model represented by Equations (4) and (5).

(4) $$\frac{dN}{dt}=\frac{Q}{1+Q}\cdot \mu_{max}\left(G_{c}\right)\cdot \mu_{LaH,H}\left(\left[LaH\right],\left[H^{+}\right]\right)\cdot N$$

(5) $$\mu_{max}\left(G_{c}\right)=a_{0}+a_{1}\cdot exp\left(-a_{2}\cdot G_{c}\right)$$

with *Gc*, the gelatine concentration; *Q*, the physiological state of the cells; $\mu_{LaH,H}\left(\left[LaH\right],\left[H^{+}\right]\right)$, the factor bringing the inhibition of growth into the model via the undissociated lactic acid concentration and the pH; $a_{0}$, $a_{1}$, and $a_{2}$ constant factors. Compared to the traditional Baranyi and Roberts [33] growth model, this model uses a more mechanistically inspired coupled-ODE method to explain growth inhibition, i.e., via the effect of the undissociated lactic acid concentration, the pH, and the gel strength. The authors concluded that the model satisfactorily predicted the effect of the gelatine concentration on the lactic acid dissociation and pH evolution and, in turn, also on the growth of the target microorganisms (in mono- and coculture). A moderate decrease in the growth rate was observed with an increasing gelatine concentration for both *Listeria innocua* and *Lactococcus lactis*, possibly explained by the increasing medium solidness imposing additional stresses on the cells.

Kapetanakou et al. [118] developed a model for the combined effect of water activity, temperature, and gelatine concentration on the growth of the fungus *Aspergillus carbonarius* in food model systems. A two-step procedure was employed during the model development, as the authors first fitted the Baranyi and Roberts [33] model to the growth data, and then fitted a polynomial secondary model (Equation (6)) to the square root of the maximum specific growth rates *µ_max_*.

(6) $$\sqrt{\mu_{max}}=a_{0}+a_{1}\cdot T+a_{2}\cdot G_{c}+a_{3}\cdot a_{w}+a_{4}\cdot T\cdot G_{c}+a_{5}\cdot T\cdot a_{w}+a_{6}\cdot G_{c}\cdot a_{w}+a_{7}\cdot T^{2}+a_{8}\cdot G_{c}{}^{2}+a_{9}\cdot a_{w}^{2}$$

with *a*_0_, *a*_1_, *a*_2_, …, and *a*_9_ the constants to be estimated; *T*, the temperature; *G_c_*, the gelatine concentration; *a_w_*, the water activity. The developed secondary model showed that the addition of gelatine caused a large decrease in $\sqrt{\mu_{max}}$, but that the structural influence was less pronounced at lower *a_w_* and *T*. The model was also validated in three commercial products, i.e., custard, marmalade, and jelly. While the model predictions agreed well with growth data on custard and marmalade, they agreed poorly with the observed data on jelly. 

Theys et al. [27] developed a model for the growth of *Salmonella* Typhimurium in function of pH, *a_w_*, and gelatine concentration in a broth model system. Similar to the two previous studies, the authors incorporated a secondary model into the primary growth model of Baranyi and Roberts [33]. This secondary model, based on the model of Ross et al. [124], is presented by Equation (7).

(7) $$\sqrt{\mu_{max}}=a_{0}\cdot \sqrt{a_{w}-a_{w,min}}\cdot \sqrt{1-10^{\left(pH_{min}-pH\right)}}\cdot \sqrt{\frac{G_{c}\cdot \mu_{liq}+a_{1}\cdot \mu_{sol}}{\left(a_{1}+G_{c}\right)\cdot \mu_{sol}}}$$

with *a*_0_ and *a*_1_ constants; *a_w_*_,*min*_ and *pH_min_*, the theoretical minimal values of *a_w_* and *pH* below which no growth occurs; *µ_liq_*, the maximum specific growth rate in liquid media (i.e., gelatine concentration equal to zero); *µ_sol_*, the maximum specific growth rate in the strongest possible solid medium (i.e., theoretical infinite gelatine concentration); *G_c_*, the gelatine concentration. In brief, the structural factor becomes equal to zero if *G_c_* equals zero, while the factor becomes equal to the ratio of *µ_sol_* and *µ_liq_* if *G_c_* is infinitely high. Based on the developed model, it was observed that the curves of the secondary model were much flatter with respect to the effect of pH and *a_w_* for the 1% and 5% gelatine concentrations than for 0% gelatine. Hence, the decrease in *µ_max_* at a higher gelatine concentration was large at a mild pH and *a_w_* conditions, while the *µ_max_* decrease with an increasing gelatine concentration was much smaller at a stressful pH and *a_w_* conditions.

In general, the three aforementioned gelatine concentration models, although exhibiting different model structures, managed to accurately describe microbial growth in the respective studied food model systems. However, those models are only valid for microbial growth in gelatine-containing food products, evidently limiting their general applicability. In order to address this issue, Aspridou et al. [103] recommended the use of a single uniform rheological parameter to describe the structure of food matrices in predictive models. The most suitable rheological parameters to include in predictive models need to be determined in future research, but might also be specific to the different food product categories. For example, the structure of more liquid food products could be described by the viscosity parameters of the Power law model of Reiner [125], while the structure of visco-elastic and solid products could be described by the storage modulus *G’*, the loss modulus *G*″, or the loss tangent tan *δ* [11,103]. It should, however, also be taken into account that rheological properties of food products can be time-dependent and that the handling of the food product (e.g., stirring, shaking) could exert a more significant influence on microbial growth than the rheological properties [27]. 

In addition to food product rheology, other food intrinsic factors related to food microstructure could also be incorporated into secondary models (e.g., fat droplet size, food matrix fat content), although more dedicated research towards the effect of those factors on microbial growth should first be conducted [126]. An extra complexity for these kinds of models, however, is related to the determination of the most suitable model structure. One could raise the question whether the food microstructural influence should be added to factors that describe the influence of other (traditional) factors on microbial growth (e.g., temperature, pH), or, whether (an) additional food microstructural factor(s) should be added to existing models. In the latter case, it would theoretically also be possible that these additional food microstructural factors are dependent on other variables which are already represented by their own factor in the traditional model (e.g., a pH-dependent influence of the gelling agent concentration on microbial growth, while a pH factor is already present in the model) [11,27]. Moreover, certain variables that are included in the microstructural factor could be dependent on other variables (e.g., a temperature-dependent rheology in a rheology-related factor). While none of these options can a priori be defined as the sole correct choice, the resulting possible complexity should be taken into account when selecting the macroscale secondary modelling approach for certain complex applications. 

### 3.2. Semi-Mechanistic Microscale Models 

While the secondary modelling approach described in Section 3.1 “Macroscale Secondary Models including Food Microstructural Factors” can result in accurate microbial growth predictions, it does not provide and/or require an extensive fundamental knowledge of the food microstructural influence on microbial dynamics. When looking at the rheology-based examples in the previous section, the microbial behaviour which is not explained by the influence of traditional factors (e.g., temperature, pH, water activity) is explained by a “black box” rheology-based factor which is not based on any physical phenomena. In reality, the influence of food rheology on microbial behaviour consists of different interacting effects, e.g., oxygen and metabolite diffusion, the mechanical distribution of water, the chemical redistribution of organic acids, and physical constraints on the mobility of microorganisms [6].

A more mechanistic modelling approach can be applied by modelling the interaction of microorganisms with the local environment based on physical phenomena. In this regard, the elementary model structure for microbial evolution is provided by Equation (8) [127].

(8) $$\frac{\partial N\left(x,y,z,t\right)}{\partial t}=\mu \left(local\;environment\right)\cdot N\left(x,y,z,t\right)$$

with *N*(*x*,*y*,*z*,*t*), the local cell density and *µ*, the specific growth rate of the microorganisms. As shown in the equation, the growth rate of the cells is dependent on the local environment, which comprises a plethora of different factors, e.g., temperature, pH, substrate concentration, metabolite concentration, and interactions with other microorganisms (i.e., same or competing species) [127]. Accurate modelling of the microbial behaviour, hence, requires an accurate modelling of the local environment of the cells. Hereby, it is important to include all factors which exert a significant influence of the microorganisms, e.g., oxygen and metabolite diffusion, the surrounding microorganisms (same species and other species), the local temperature, and local physicochemical conditions such as pH and a_w_. In this regard, partial differential equations (PDEs), describing changes in variables as a function of space and time, are often introduced into the predictive models [3]. This methodology also allows the inclusion of changes in environmental factors over time in a straightforward way. This can be useful to include the time-dependent nature of food structure into models; it is, for example, known that the rheological properties of food products can change over time [27]. Taking the local conditions into account leads to the development of microscale models, rather than the macroscale models (i.e., focussing on total cell populations and macroscopic food properties) discussed in the previous section. Since microscale models need to deal with a high level of detail (e.g., spatial and microbial heterogeneity), they have a high complexity, possibly leading to significant computational costs [128]. It should also be noted that no fully mechanistic models for microbial dynamics exist to date, since (i) the current microbiological knowledge is too limited for fully mechanistic relations and (ii) empiric relations are sometimes used to describe some environmental conditions in order to save computational efforts. Consequently, most predictive models are semi-mechanistic, meaning that they contain some mechanistic information in their structure and physically measurable parameters [128]. 

Dens and Van Impe [119] proposed a general modelling approach to take spatial heterogeneity in structured foods into account. While the model was solely based on model simulations and, hence, not based on experiments in structured model systems, the study was included in this review because of its importance for models developed in later studies. In brief, the authors extended a previously developed mixed population growth model for homogeneous food products, unifying the growth model of Baranyi and Roberts [33] and the Lotka–Volterra model [129] for two-species competition (i.e., in this case, *Escherichia coli* and *Lactobacillus plantarum*), and introducing a (two-dimensional) space-dependency into the model. The general model structure is shown by Equations (9) and (10), with the respective first terms representing bacterial growth via the combined Baranyi and Roberts [33] and Lotka–Volterra [129] model, and the respective second terms representing the biomass transport via diffusion.

(9) $$\frac{\partial N_{1}\left(x,y,t\right)}{\partial t}=\mu_{1}\left(x,y,t\right)\cdot N_{1}\left(x,y,t\right)+D\cdot \nabla^{2}N_{1}\left(x,y,t\right)$$

(10) $$\frac{\partial N_{2}\left(x,y,t\right)}{\partial t}=\mu_{2}\left(x,y,t\right)\cdot N_{2}\left(x,y,t\right)+D\cdot \nabla^{2}N_{2}\left(x,y,t\right)$$

The complete form of these model equations is represented by Equations (11) and (12).

(11) $$\frac{\partial N_{1}\left(x,y,t\right)}{\partial t}=\mu_{max,1}\cdot \frac{Q_{1}\left(x,y,t\right)}{1+Q_{1}\left(x,y,t\right)}\cdot \frac{N_{1}\left(x,\;y,t\right)}{N_{max,1}}\cdot \left(N_{max,1}-N_{1}\left(x,y,t\right)-\alpha_{1,2}\cdot N_{2}\left(x,y,t\right)\right)+D\;\cdot \left(\frac{\partial^{2}N_{1}\left(x,y,t\right)}{\partial x^{2}}+\frac{\partial^{2}N_{1}\left(x,y,t\right)}{\partial y^{2}}\right)$$

(12) $$\frac{\partial N_{2}\left(x,y,t\right)}{\partial t}=\mu_{max,2}\cdot \frac{Q_{2}\left(x,y,t\right)}{1+Q_{2}\left(x,y,t\right)}\frac{N_{2}\left(x,\;y,t\right)}{N_{max,2}}\cdot \left(N_{max,2}-N_{2}\left(x,y,t\right)-\alpha_{2,1}\cdot N_{1}\left(x,y,t\right)\right)+D\;\cdot \left(\frac{\partial^{2}N_{2}\left(x,y,t\right)}{\partial x^{2}}+\frac{\partial^{2}N_{2}\left(x,y,t\right)}{\partial y^{2}}\right)$$

with *N*_1_ and *N*_2_, the cell densities of the two bacterial species; *Q*_1_ and *Q*_2_, the internal physiological state of both species used to describe the lag phase; *µ_max_*_,1_ and *µ_max_*_,2_, the maximum specific growth rate of both species; *N_max_*_,1_ and *N_max_*_,2_, the maximum population density of both species when grown in monoculture; *α_1,2_* and α_2,1_, the interaction coefficients measuring the effects of species one on species two and vice versa; *D*, the cell diffusivity; $\nabla^{2}$, the diffusive operator. For their simulations, the authors assumed a 10 by 10 cm agar gel, 1 mm thick, to allow a two-dimensional model. Space was considered as a grid of lattice sites, with each site being assumed homogeneous. The biomass mass transfer was taken into account via the diffusion law, while the food structure was included via factor *D*, a measure for the firmness of the food. Factor *D* was assumed infinite for very fluid foods and zero for solid foods in which no movement of microorganisms is possible. In their concluding remarks, Dens and Van Impe [119] state that, while their model simulations should not be regarded as accurate, the main message of their work is that an extended model structure taking space into account is necessary to model microbial growth in structured food environments. In later years, other authors have indeed used this approach to model microbial growth in structured environments, as shown by the two following examples of Noriega et al. [98,115] and De Bonis and Ruocco [120].

Noriega et al. [98,115] developed a predictive model for *L. innocua* growth in solid or paste foods, taking into account oxygen diffusion limitations. Three different dissolved oxygen concentrations were investigated in solidified broth systems, i.e., (i) aerobic conditions (7.6–8.0 mg/L), (ii) hypoxic conditions (0.2–2.6 mg/L), and (iii) anoxic conditions (<0.01 mg/L). In brief, the used modelling approach was a combination of the logistic Riccati equation for microbial growth (Equation (13) [130]), on the one hand, and oxygen (Equation (14)) and substrate (Equation (15)) mass balances, on the other.

(13) $$\frac{dN}{dt}=K\cdot N\cdot \left(1-\tau \cdot N\right)$$

(14) $$\frac{\partial N}{\partial t}=D\cdot \frac{\partial^{2}N}{\partial z^{2}}+\frac{dN}{dt}$$

(15) $$\frac{\partial C_{O_{2}}}{\partial t}=D_{O_{2}}\cdot \frac{\partial^{2}C_{O_{2}}}{\partial z^{2}}+\frac{dC_{O_{2}}}{dt}\;$$

with *D*, the cell diffusivity; $D_{O_{2}}$, the oxygen diffusion rate; $C_{O_{2}}$, the oxygen concentration; *K* and *τ*, kinetic parameters obtained in liquid media, with *τ* being a function of the oxygen concentration. The authors concluded that the used approach of combining kinetic parameters as a function of oxygen concentration, obtained in liquid medium with the assumption of oxygen as a limiting substrate for cell growth, resulted in accurate model predictions for structured media.

De Bonis and Ruocco [120] developed a mathematical 3D model of a structured leafy product to simulate *Escherichia coli* growth in fresh iceberg lettuce during handling and storage. In brief, the model combined general first-law equations for heat transfer (Equation (16)), biomass kinetics and transfer (Equation (17)), primary bacterial growth kinetics (Equation (18) [131]), and secondary temperature-dependent bacterial growth kinetics (Equation (19) [132]). Structural features of the food were taken into account by a multiplicity of inter-leaf contact points and insulating air pockets, which influence microbial growth.

(16) $$\rho \cdot c_{P}\cdot \frac{\partial T}{\partial t}=k_{S}\cdot \nabla^{2}T$$

(17) $$\frac{\partial N}{\partial t}=D\cdot \nabla^{2}N+\frac{dN}{dt}$$

(18) $$\frac{dN}{dt}=\frac{Q}{1+Q}\cdot \mu_{max}\cdot \left(1-\frac{N}{N_{max}}\right)\cdot N$$

(19) $$\sqrt{\mu_{max}}=b\cdot \left(T-T_{min}\right)$$

with *ρ*, the substrate density; *c_p_*, the substrate specific heat capacity; *T*, the temperature; *k_S_*, the substrate conductivity; *D*, the cell diffusivity in the substrate; *µ_max_*, the maximum specific growth rate; *Q*, the physiological state of the cells; *N_max_*, the maximum cell density; *b*, the kinetic parameter of the Ratkowsky equation; *T_min_*, the reference temperature. The predicted thermal profiles were validated in real iceberg lettuce samples, showing that the model predicted temperature evolution nicely at all considered depths. The microbial behaviour was not experimentally validated, but depended on general data on microbial generation and diffusion. A value for bacterial (constant temperature) diffusivity was obtained from literature for this purpose. In general, the main advantage of the model of De Bonis and Ruocco [120] is that it is a general engineering tool which stems from the integration of partial differential equations that describe heat and mass transfer. The model allows the prediction of local and volume-averaged bacterial cell growth with proper accuracy, and both in function of the initial contamination and the operating thermal regime of the product.

The previously discussed approaches can be classified as grid-based or biomass-based models (BbM), as the cell density in a small volume unit of the food product was used as the basic unit to model microbial growth. A further step towards more accurate models would be to include direct intercellular reactions by using individual cells as the basic model units in individual-based models (IbMs) [128]. IbMs provide realistic bacterial dynamics and can be designed to include accurate descriptions of complex micro-structures and environments [133]. Over the last two decades, IbMs have shown increasing potential for the modelling of microbial behaviour due to the development of specialised software [134]. Examples of IbM software tools for predictive microbiology include BacSim [135], INDISIM [136], MICRODIMS [137,138], BSim [133], and iDynoMiCS [139]. The potential of IbMs to predict microbial dynamics in complex systems (i.e., complex environment and/or complex microflora), even including the complex behaviour in microbial biofilms, is a major advantage for modelling applications in structured food products [140]. While an in-depth explanation of IbMs lies outside the scope of this study, it is worth mentioning the most relevant example of an IbM approach applied to microbial growth on (i.e., surface growth) structured food products.

Ferrier et al. [121] and Augustin et al. [122] developed an IBM approach to describe the behaviour of a small number of *Listeria monocytogenes* cells contaminating the surface of smear soft cheese and vacuum-packed cold-smoked salmon. Microscale models describing the local pH and a_w_ over the food surface were constructed based on microelectrode measurements. These models were combined with the IBM approach to simulate the stochastic growth of the bacteria on the product; simulations were also validated on real cheese and salmon samples. On the one hand, the authors concluded that, for no-growth or poor-growth situations (i.e., a small number of cells), the accuracy of their coupled IBM approach surpassed the classical macroscale approach. On the other hand, the results of the two approaches were similar when assessing the impact of changes in control measures influencing the growth of the bacteria. Therefore, the IbM approach was mainly useful to predict single-cell growth probability of foodborne pathogens contaminating food with a small number of cells. Nevertheless, more microenvironmental factors, as well as the interaction of *L. monocytogenes* with background microflora, should be added to the model in order to further increase its accuracy.

## 4. Inactivation Models Incorporating Food Microstructure

Due to the long history of thermal processing as a means of food preservation in the food industry, most inactivation models that incorporate the food microstructural influence are thermal inactivation models, which are, hence, the main focus of this section. Although predictive inactivation models have been developed for novel non-thermal technologies, these are in most cases solely based on experiments in liquid laboratory media (e.g., for high pressure processing [141]). 

Similar to the microbial growth models, all reported thermal inactivation models which include food microstructure can be classified into two categories, i.e., (i) macroscale secondary models including food microstructural factors and (ii) semi-mechanistic microscale models. Table 3 provides an overview of the most relevant models of the two categories. A more detailed description of the respective models can be found in the following sections.

### 4.1. Macroscale Secondary Models Including Food Microstructural Factors

To the best knowledge of the authors, the only existing macroscopic thermal inactivation models that incorporate food microstructural factors are based on fat content and water mobility. Fat content is often categorised as a food compositional factor, but since fat content also determines the microstructural characteristics of emulsion-type foods, the classification of fat content as a food microstructural factor is justified in some cases. While some models were developed specifically for certain food products types (e.g., for poultry with different fat content [19]), this section is focussed on inactivation models developed in artificial food model systems, because this approach fits more into the general predictive microbiology mindset. 

Chhabra et al. [142] were among the first to develop a fat content-based inactivation model using food model systems, based on homogenised milk. They developed a model for the inactivation of *L. monocytogenes* in function of the fat content, pH, and processing temperature. The developed model was a modified Gompertz equation, as shown in Equation (20), including parameters *S_L_*, *k_max_*, and *N_red_*, characterising the shoulder phase, the maximum inactivation rate, and the overall change in the number of survivors, respectively. These parameters depended on the fat content, pH, and processing temperature, as indicated in Equations (21)–(23).

(20) $$\log N=N_{red}\cdot e^{-e^{\left(S_{L}+k_{max\cdot }t\right)}}-N_{red}\cdot e^{-e^{\left(S_{L}\right)}}$$

(21) $$S_{L}=a_{0}+a_{1}\cdot C_{F}+a_{2}\cdot pH+a_{3}\cdot T+a_{1,2}\cdot C_{F}\cdot pH+a_{1,3}\cdot C_{F}\cdot T+a_{2,3}\cdot pH\cdot T+a_{1,2,3}\cdot C_{F}\cdot pH\cdot T$$

(22) $$k_{max}=b_{0}+b_{1}\cdot C_{F}+b_{2}\cdot pH+b_{3}\cdot T+b_{1,2}\cdot C_{F}\cdot pH+b_{1,3}\cdot C_{F}\cdot T+b_{2,3}\cdot pH\cdot T+b_{1,2,3}\cdot C_{F}\cdot pH\cdot T\;$$

(23) $$N_{red}=c_{0}+c_{1}\cdot C_{F}+c_{2}\cdot pH+c_{3}\cdot T+c_{1,2}\cdot C_{F}\cdot pH+c_{1,3}\cdot C_{F}\cdot T+c_{2,3}\cdot pH\cdot T+c_{1,2,3}\cdot C_{F}\cdot pH\cdot T$$

with *C_F_*, the fat content parameter; *a*_0_−*c*_1,2,3_, constant factors. Theoretically, the model could have also been extended to include terms of higher order. It should, however, be noted that not all parameters were deemed significant. For instance, the shoulder region of the inactivation was only affected by the pH, while the death rate was only affected by the temperature and fat content. It was also shown that, as temperature increased, there was a decrease in heat resistance due to the presence of milkfat. However, modified Gompertz models for inactivation are, apart from some mathematical limitations, characterised by two major modelling problems, i.e., (i) *N*(*t* = 0) is not equal to *N*(0) in the static version of the model and (ii) there is no explicit dependency on *N*(0) in the dynamic version of the model, which should be avoided [61,146].

Santillana Farakos et al. [143] used whey protein powder model systems to develop a predictive model for the thermal inactivation of *Salmonella* in low-moisture foods in function of temperature, a_w_, and water mobility. The water mobility of the different model systems was acquired by a pH adjustment and heat denaturation, and equilibrated to a_w_ levels between 0.19 ± 0.03 and 0.54 ± 0.02. The specific water mobility values were determined by means of wide-line proton-NMR (Nuclear Magnetic Resonance). Four different models were fitted to the inactivation data, but the Weibull model (Equation (24) [147]) was selected for secondary modelling, because it best described the data over all temperatures.

(24) $$\log N=\log N_{0}-{\left(\frac{t}{\delta }\right)}^{\beta }$$

with *N*_0_, the initial cell population; *δ*, the scale parameter representing the treatment time (in min) required to for the first decimal log reduction in the cell population; *β*, the shape factor value. In order to develop secondary models, the significance of the temperature, a_w_, and water mobility on log *δ* and log *β* was assessed. The temperature was deemed to be a significant influencing factor on both Weibull parameters, while a_w_ was only deemed to be a significant influencing factor on log *δ*. The water mobility, however, being the only investigated food microstructural factor, did not exert a significant influence on any of the parameters. Hence, the developed model, as shown in Equations (25) and (26), did not explicitly incorporate the influence of food microstructure.

(25) $$\log\delta =-0.10T-4.34a_{w}+9.91$$

(26) logβ=−0.006T

Nevertheless, the model achieved acceptable predictions in different real food products, i.e., low-fat cocoa powder, low-fat peanut meal, non-fat dry milk, wheat flour, and whey protein.

More recently, Trimble et al. [144] adapted the aforementioned model of Santillana Farakos et al. [143] to include the influence of fat content on *Salmonella* inactivation in low-water-activity foods, using whey protein–peanut oil powders as model systems. For this purpose, they eliminated the correlation between *δ* and *β* by using a fixed value of *β* = 0.3644. This resulted in a secondary model expressing a significant influence of the temperature, fat content (*C_F_*), and water activity on log *δ*, as shown in Equation (27).

(27) $$\log\delta =22.90-0.167T+0.051C_{F}-4.38a_{w}$$

The adapted model was validated in fat-containing low-*a_w_* foods, i.e., toasted oats, animal crackers, chia seed powder, and natural peanut butter. A slight underestimation of thermal inactivation by the model was reported, but the model was still deemed successful for the prediction of *Salmonella* survival in low-*a_w_* foods. In their final remarks, Trimble et al. [144] suggested that the model could still be expanded to include a wider fat content and/or temperature range and a dependency on fat type. In general, it should, however, be mentioned that the Weibull model is characterized by two disadvantages. First of all, the frequency distribution of the viability of bacterial cells, the concept on which the Weibull model is based, is difficult to interpret and to validate experimentally [148]. Secondly, the Weibull model lacks a suitable differential model form. Due to the frequent use of ODEs and PDEs in predictive modelling frameworks that include food microstructure, the developed model strategy may, hence, have limited applicability for more complex microbial inactivation cases.

### 4.2. Semi-Mechanistic Microscale Models

An approach which has been extensively used to implicitly incorporate food microstructure into predictive thermal inactivation models is linking the bacterial inactivation model to (space- and time-dependent) heat and mass transfer models. This methodology is especially suitable for thermal inactivation because structural food properties tend to change during thermal processing, e.g., the viscosity of liquid foods which decreases with an increasing temperature or certain foods which further solidify during frying [149,150]. Zanoni et al. [55] were among the first to adopt this approach, although for experiments carried out in a real food product (i.e., bologna sausage). They combined the Whiting et al. [151] inactivation model with a heat and mass transfer model validated for bologna sausage cooking. A similar approach was later on conducted in food model systems by researchers from the School of Chemical Engineering (University of Birmingham) and Institute of Food Research (Reading Laboratory) (e.g., [112,114]). These authors conducted thermal inactivation experiments in agar cylinders and modelled both the heat transfer and thermal inactivation kinetics, the latter by means of experimentally obtained D and z-values. These studies marked an important step in the thermal inactivation model development because the used approach could be used with food products of different geometry or thermal conductivity, and for different bacteria.

Over the last decade, computer modelling techniques for heat and mass transfer have become more and more common when modelling treatments of different traditional and novel thermal processing technologies, e.g., beverage pasteurization [152], agitated retort heating [153], microwave heating [154], continuous deep frying [155], and radio frequency heating [156]. In brief, such techniques involve solving the heat and mass transfer equations with applied initial and boundary conditions using either (i) theoretical numerical finite difference and finite elements solutions or (ii) a computational fluid dynamics (CFD) approach [157]. While such models would be suitable to be coupled to microbial thermal inactivation models, this approach has only been scarcely employed for these industry-relevant processes [145].

Hamoud-Agha et al. [113] investigated the thermal inactivation of *Escherichia coli* K12 in calcium alginate gels during microwave processing. The inactivation model of Geeraerd et al. [61], including a Bigelow-type temperature dependency of the inactivation rate, was coupled to heat transfer and Maxwell’s equations into a 3D finite elements model under dynamic heating conditions. By providing space-dependent predictions, the model was able to handle the thermal heterogeneity inherent to microwave treatments and the resulting differences in inactivation efficiency between the different locations within samples. Consequently, Hamoud-Agha et al. [113] demonstrated the reliability of the coupled modelling approach which links microbial inactivation models to heat transfer models. 

A similar approach was used by Albuquerque et al. [145] for the thermal inactivation of *Escherichia coli* K12 in pre-packed ground beef in water baths programmed to deliver different heating rates to the product. The authors coupled a 3D-CFD and heat transfer finite elements model to the inactivation model of Geeraerd et al. [61], including a Bigelow-type temperature dependent inactivation rate. Even though the large heating rates caused large temperature gradients and heterogeneous inactivation distributions over the samples, there was still satisfactory agreement between model predictions and experimental data. Moreover, and most relevant for this review, the model was able to handle the typical microstructural complexity of the real food product under study.

The studies of Hamoud-Agha et al. [113] and Albuquerque et al. [145], hence, demonstrate that coupling microbial thermal inactivation models to heat and mass transfer models is a promising, and probably the most optimal, approach to develop microbial (thermal) inactivation models which take the food microstructural influence on microbial inactivation into account. The fact that the other possible method to include the food microstructural effect into predictive models (i.e., macroscale secondary models) has so far solely relied on less optimal model types for inactivation, such as the modified Gompertz and Weibull model, strengthens this conclusion. Similar to microbial growth, the accuracy of the microscale methods could be further improved by including an IbM approach. The inactivation of small cell populations is often characterised by a high variability in inactivation behaviour, originating from individual cell heterogeneity [158,159]. However, an appropriate theoretical IbM approach for modelling of the variability in individual cell heterogeneity during the inactivation process has not been developed thus far [160]. 

## 5. Conclusions

While the influence of food microstructure on microbial dynamics was for the largest part neglected in the early days of predictive microbiology, significant progress on the subject has been achieved during the last two decades. Both for microbial growth and thermal inactivation, two general model types can be distinguished in the scientific literature, i.e., (i) macroscale secondary models including food microstructural factors and (ii) microscale semi-mechanistic models. These model types have benefited from the introduction of advanced mathematical modelling techniques and the increased usage of artificial food model systems to collect experimental data (i.e., rather than real food products). 

In general, both the macroscopic secondary models and the semi-mechanistic microscale models have shown their potential as predictive modelling tools/frameworks when predicting microbial growth and inactivation in/on structured food products. The selection of one approach over another, or a combination of the two approaches, should ideally depend on the specific application, the required accuracy of the model, and the available computing power. Specifically for the macroscopic secondary models, other food intrinsic factors related to food microstructure could also be taken into account in addition to food product rheology (growth) and fat content (thermal inactivation), although more dedicated research towards the effect of those additional factors on microbial dynamics should first be conducted. Moreover, a lot of progress is still to be made for microbial inactivation via non-thermal technologies, as models including the food microstructural influence on microbial inactivation for those technologies are to date virtually non-existent. Specifically for the microscale semi-mechanistic models, the development of models taking more aspects of the complex microstructural and microbial environment into account forms an interesting research opportunity.

## Figures and Tables

**Table 1:** Examples of studies (ordered chronologically) in which predictive models only valid for specific food products were developed.

Year	Type	Microorganism(s)	Food Product	Ref.
1981	Growth	*Clostridium botulinum*	Pork slurry	[82]
1985	Growth	Various background microflora	Beef	[86]
1990	Growth	*Clostridium botulinum*	Fish filets	[66]
1992	Growth	*Salmonella* Typhimurium	Beef	[70]
1996	Growth	Various background microflora	Cut endive	[85]
1997	Inactivation	*Enterococcus faecium*	Bologna sausage	[55]
1998	Growth	Various background microflora	Sausage	[65]
1998	Growth	Various background microflora	Beef	[71]
1999	Inactivation	*Salmonella enteritidis*	Tarama salad	[93]
1999	Inactivation	*Enterobacter sakazakii*	Bovine whole milk	[94]
1999	Growth	*Salmonella* Typhimurium	Cooked chicken breast	[79]
1999	Growth	*Pseudomonas* spp. and *Shewanella putrefaciens*	Fresh bogue fish	[83]
2003	Inactivation	*Staphylococcus aureus*	Surimi seafood sticks	[89]
2013	Inactivation	*Salmonella*	Ground chicken	[91]
2014	Inactivation	*Listeria monocytogenes*	Ground turkey	[92]
2016	Inactivation	*Salmonella*	Tree nuts	[96]
2018	Growth	*Bacillus cereus*	Cooked spinach	[73]
2018	Growth	*Bacillus cereus* (spores)	Cooked beans	[74]
2018	Growth	*Escherichia coli*	Mascarpone cheese	[76]
2018	Growth	*Weissella viridescens*	Vacuum-packaged ham	[78]
2018	Growth	*Escherichia coli*	Korean rice cake	[80]
2018	Inactivation	*Escherichia coli*	Ground chicken	[97]
2019	Growth	*Staphylococcus aureus*	Egg products	[68]
2019	Growth	*Vibrio parahaemolyticus*	Korean raw crab marinated in soy sauce	[69]
2019	Inactivation	*Listeria monocytogenes*	Gilthead sea bream fillets	[87]
2019	Growth	*Bacillus cereus*	Cooked rice	[72]
2019	Growth/inactivation	*Listeria monocytogenes*	Fish balls	[90]
2019	Growth	*Bacillus cereus* (spores)	Cooked pasta	[75]
2019	Growth	*Clostridium perfringens*	Roasted chicken and braised beef	[77]
2019	Growth	*Aeromonas hydrophila*	Lettuce	[81]
2019	Inactivation	*Salmonella*	Infant formula	[95]
2020	Growth	*Brochothrix thermosphacta*, *Leuconostoc gelidum* and *Pseudomonas* spp.	Minced pork	[67]
2020	Inactivation	*Salmonella* Thompson	Iceberg lettuce	[88]
2020	Growth	*Salmonella* Reading and lactic acid bacteria	Iceberg lettuce	[84]

**Table 2:** Overview of the most relevant growth models incorporating food microstructure.

Macroscale Secondary Models			
Model Description	Microstructural Factors	Non-Microstructural Factors	Ref.
*Listeria innocua* and *Lactococcus lactis* growth (mono- and co-culture) in a gelled system	Gelatine concentration	Undissociated lactic acid concentration, pH, physiological state of the cells (for lag phase)	[24]
*Aspergillus carbonarius* growth in broth	Gelatine concentration	Temperature, water activity, physiological state of the cells (for lag phase)	[118]
*Salmonella* Typhimurium growth in broth	Gelatine concentration	Water activity, pH, physiological state of the cells (for lag phase)	[27]
**Semi-Mechanistic Microscale Models**			
**Model Description**	**Included Environmental Factors**		**Ref.**
Mixed population growth model for homogeneous food products, with 2-dimensional space dependency	Food structure (via firmness of the food), biomass transport (via diffusion)		[119]
*Listeria innocua* growth in solid or paste foods	Dissolved oxygen concentration (and diffusion), biomass transport (via diffusion)		[98,115]
*Escherichia coli* growth in a (3D)-structured leafy product during handling and storage	Temperature/heat transfer, biomass transfer (via diffusion), leafy structure (via inter-leaf contact points and entrapped air pockets)		[120]
*Listeria monocytogenes* growth on the surface of smear soft cheese and vacuum-packed cold-smoked salmon	Local pH, local water activity, temperature, structural environment (e.g., hollows, crests)		[121,122]

**Table 3:** Overview of the most relevant thermal inactivation models incorporating food microstructure.

**Macroscale Secondary Models**			
**Model Description**	**Microstructural Factors**	**Non-Microstructural** **Factors**	**Ref.**
*Listeria monocytogenes* inactivation in homogenised milk model systems	Fat content	Temperature, pH	[142]
*Salmonella* inactivation in whey protein powder model systems	Water mobility	Temperature, water activity	[143]
*Salmonella* inactivation in whey protein–peanut oil powders model systems	Fat content	Temperature, water activity	[144]
**Semi-Mechanistic Microscale Models**			
**Model Description**	**Included Environmental Factors**		**Ref.**
*Escherichia coli* K12 thermal inactivation (microwave) in calcium alginate gels	Local temperature (via microwave dielectric heating and heat transfer, taking thermophysical properties of the gels into account)		[113]
*Escherichia coli* K12 thermal inactivation in pre-packed ground beef in water baths	Fluid flow in water bath, local temperature (via heat transfer, taking thermophysical properties of the ground beef into account)		[145]

## Data Availability

No new data were created or analysed in this study. Data sharing is not applicable to this article.

## References

[1] McMeekin T.A., Olley J., Ratkowsky D.A., Ross T. (2002). Predictive microbiology: Towards the interface and beyond. Int. J. Food Microbiol..

[2] Stavropoulou E., Bezirtzoglou E. (2019). Predictive modeling of microbial behavior in food. Foods.

[3] Lopatkin A.J., Collins J.J. (2020). Predictive biology: Modelling, understanding and harnessing microbial complexity. Nat. Rev. Microbiol..

[4] Pérez-Rodríguez F., Carrasco E., Pradhan A.K., Sant’Ana A.S., Valdramidis V.P., Valero A. (2019). Special issue on 10th international conference of predictive modelling in foods: Towards a new paradigm in predictive microbiology. Int. J. Food Microbiol..

[5] McDonald K., Sun D.-W. (1999). Predictive food microbiology for the meat industry: A review. Int. J. Food Microbiol..

[6] Heertje I. (2014). Structure and function of food products: A review. Food Struct..

[7] Aguilera J.M. (2005). Why food microstructure?. J. Food Eng..

[8] Bhopatkar D., Hamaker B.R., Campanella O.H., Bhandari B., Roos Y.H. (2012). Micro to macro level structures of food materials. Food Materials Science and Engineering.

[9] Ubbink J., Burbridge A., Mezzenga R. (2008). Food structure and functionality: A soft matter perspective. Soft Matter.

[10] Wilson P.D.G., Brocklehurst T.F., Arino S., Thualt D., Jakobsen M., Lange M., Farkas J., Wimpenny J.W.T., Van Impe J.F. (2002). Modelling microbial growth in structured foods: Towards a unified approach. Int. J. Food Microbiol..

[11] Verheyen D., Bolívar A., Pérez-Rodríguez F., Baka M., Skåra T., Van Impe J.F. (2018). Effect of food microstructure on growth dynamics of *Listeria monocytogenes* in fish-based model systems. Int. J. Food Microbiol..

[12] Wimpenny J.W.T., Leistner L., Thomas L.V., Mitchell A.J., Katsaras K., Peetz P. (1995). Submerged bacterial colonies within food and model systems: Their growth, distribution and interactions. Int. J. Food Microbiol..

[13] Verheyen D., Xu X.M., Govaert M., Baka M., Van Impe J.F. (2019). Food microstructure and fat content affect growth morphology, growth kinetics, and preferred phase for cell growth of *Listeria monocytogenes* in fish-based model systems. Appl. Environ. Microbiol..

[14] Mertens L., Geeraerd A.H., Dang T.D.T., Vermeulen A., Serneels K., Van Derlinden E., Cappuyns A.M., Moldenaers P., Debevere J., Devlieghere F. (2009). Design of an experimental viscoelastic food model system for studying *Zygosaccharomyces bailii* spoilage in acidic sauces. Appl. Environ. Microbiol..

[15] Baka M., Noriega E., Van Langendonck K., Van Impe J.F. (2016). Influence of food intrinsic complexity on *Listeria monocytogenes* growth in/on vacuum-packed model systems at suboptimal temperatures. Int. J. Food Microbiol..

[16] Pérez-Rodríguez F., Valero A. (2013). Predictive Microbiology in Foods.

[17] Theys T. (2009). Modelling the (Boundaries of) Microbial Growth in Structured Media: Effect of pH, Water Activity and Gelatin on the Growth of *Salmonella* Typhimurium. Ph.D. Thesis.

[18] Velliou E.G., Noriega E., Van Derlinden E., Mertens L., Boons K., Geeraerd A.H., Devlieghere F., Van Impe J.F. (2013). The effect of colony formation on the heat inactivation dynamics of *Escherichia coli* K12 and *Salmonella* typhimurium. Food Res. Int..

[19] Juneja V.K., Eblen B.S., Marks H.M. (2001). Modeling non-linear survival curves to calculate thermal inactivation of *Salmonella* in poultry of different fat levels. Int. J. Food Microbiol..

[20] Murphy R.Y., Marks B.P., Johnson E.R., Johnson M.G. (2000). Thermal inactivation kinetics of *Salmonella* and *Listeria* in ground chicken breast meat and liquid medium. J. Food Sci..

[21] Verheyen D. (2020). Micro- and Macroscopic Investigation of the Food Microstructural Influence on Microbial Dynamics: Case Study in/on Fish Products. Ph.D. Thesis.

[22] Smet C., Noriega E., Van Mierlo J., Valdramidis V.P., Van Impe J.F. (2015). Influence of the growth morphology on the behaviour of *Salmonella* Typhimurium and *Listeria monocytogenes* under osmotic stress. Food Res. Int..

[23] Robins M.M., Wilson P.D.G. (1994). Food structure and microbial growth. Trends Food Sci. Technol..

[24] Antwi M., Bernaerts K., Van Impe J.F., Geeraerd A.H. (2007). Modelling the combined effects of structured food model system and lactic acid on *Listeria innocua* and *Lactococcus lactis* growth in mono- and coculture. Int. J. Food Microbiol..

[25] Skandamis P.N., Jeanson S. (2015). Colonial vs. Planktonic type of growth: Mathematical modelling of microbial dynamics on surfaces and in liquid, semi-liquid and solid foods. Front. Microbiol..

[26] Buchanan R.L. (1993). Predictive food microbiology. Trends Food Sci. Technol..

[27] Theys T.E., Geeraerd A.H., Verhulst A., Poot K., Van Bree I., Devlieghere F., Moldenaers P., Wilson D., Brocklehurst T., Van Impe J.F. (2008). Effect of pH, water activity and gel micro-structure, including oxygen profiles and rheological characterization, on the growth kinetics of *Salmonella* Typhimurium. Int. J. Food Microbiol..

[28] Genigeorgis C., Martin S., Franti C.E., Riemann H. (1971). Initiation of Staphylococcal growth in laboratory media. Appl. Microbiol..

[29] Nixon P.A. (1971). Temperature integration as a means of assessing storage conditions. Report on Quality in Fish Products, Seminar No. 3.

[30] Spencer R., Baines C.R. (1964). The effect of temperature on the spoilage of wet fish: I. Storage at constant temperature between −1 °C and 25 °C. Food Technol. Champ..

[31] Gompertz B. (1825). On the nature of the function expressive of the law of human mortality, and on a new mode of determining the value of life contingencies. Philos. Trans. R. Soc. Lond..

[32] Zwietering M.H., Jongenburger I., Rombouts F.M., van’t Riet K. (1990). Modeling of the bacterial growth curve. Appl. Environ. Microbiol..

[33] Baranyi J., Roberts T.A. (1994). A dynamic approach to predicting bacterial growth in food. Int. J. Food Microbiol..

[34] Bhaduri S., Turner-Jones C.O., Buchanan R.L., Phillips J.G. (1994). Response surface model of the effect of pH, sodium chloride and sodium nitrite on growth of *Yersinia enterocolitica* at low temperatures. Int. J. Food Microbiol..

[35] George S.M., Richardson L.C.C., Peck M.W. (1996). Predictive models of the effect of temperature, pH and acetic and lactic acid on the growth of *Listeria monocytogenes*. Int. J. Food Microbiol..

[36] Ng T.M., Schaffner D.W. (1997). Mathematical models for the effects of pH, temperature, and sodium chloride on the growth of *Bacillus stearothermophilus* in salty carrots. Appl. Environ. Microbiol..

[37] Sutherland J.P., Bayliss A.J., Braxton D.S. (1995). Predictive modelling of growth of *Escherichia coli* O157:H7: The effects of temperature, pH and sodium chloride. Int. J. Food Microbiol..

[38] Augustin J.-C., Carlier V. (2000). Modelling the growth rate of *Listeria monocytogenes* with a multiplicative type model including interactions between environmental factors. Int. J. Food Microbiol..

[39] Le Marc Y., Huchet V., Bourgeois C.M., Guyonnet J.P., Mafart P., Thuault D. (2002). Modelling the growth kinetics of *Listeria* as a function of temperature, pH and organic acid concentration. Int. J. Food Microbiol..

[40] Panagou E.Z., Skandamis P.N., Nychas G.-J.E. (2003). Modelling the combined effect of temperature, pH and a_w_ on the growth rate of *Monascus ruber*, a heat-resistant fungus isolated from green table olives. J. Appl. Microbiol..

[41] Little C.L., Knøchel S. (1994). Growth and survival of *Yersinia enterocolotica*, *Salmonella* and *Bacillus cereus* in Brie stored at 4, 8 and 20 °C. Int. J. Food Microbiol..

[42] Meldrum R.J., Brocklehurst T.F., Wilson D.R., Wilson P.D.G. (2003). The effects of cell immobilization, pH and sucrose on the growth of *Listeria monocytogenes* Scott A at 10 °C. Food Microbiol..

[43] Ongeng D., Ryckeboer J., Vermeulen A., Devlieghere F. (2007). The effect of micro-architectural structure of cabbage substratum and or background bacterial flora on the growth of *Listeria monocytogenes*. Int. J. Food Microbiol..

[44] Esty J.R., Meyer K.F. (1922). The heat resistance of the spore of *B. botulinus* and allied anaerobes XI. J. Infect. Dis..

[45] Bigelow W.D. (1921). The logarithmic nature of thermal death time curves. J. Infect. Dis..

[46] Bevilacqua A., Speranza B., Sinigaglia M., Corbo M.R. (2015). A focus on the death kinetics in predictive microbiology: Benefits and limits of the most important models and some tools dealing with their application in foods. Foods.

[47] Desriac N., Vergos M., Achberger V., Coroller L., Couvert O. (2019). Predicting heat process efficiency in thermal processes when bacterial inactivation is not log-linear. Int. J. Food Microbiol..

[48] Ball C.O., Olson F.C.W. (1957). Sterilization in Food Technology: Theory, Practice and Calculation.

[49] Garrett E.R. (1956). Prediction of stability in pharmaceutical preparation II. Vitamin stability in liquid multivitamin preparations. J. Am. Pharm. Assoc..

[50] Levine S. (1956). Determination of the thermal death rate of bacteria. Food Res..

[51] Davey K.R. (1993). Linear-Arrhenius models for bacterial growth and death and vitamin denaturations. J. Ind. Microbiol..

[52] Cerf O., Davey K.R., Sadoudi A.K. (1996). Thermal inactivation of bacteria—A new predictive model for the combined effect of three environmental factors: Temperature, pH and water activity. Food Res. Int..

[53] Davey K.R., Lin S.H., Wood D.G. (1978). The effect of pH on continuous high-temperature/short-time sterilization of liquid. Am. Inst. Chem. Eng. J..

[54] Blackburn C.d.W., Curtis L.M., Humpheson L., Billon C., McClure P.J. (1997). Development of thermal inactivation models for *Salmonella enteritidis* and *Escherichia coli* O157:H7 with temperature, pH and NaCl as controlling factors. Int. J. Food Microbiol..

[55] Zanoni B., Peri C., Garzaroli C. (1997). A dynamic mathematical model of the thermal inactivation of *Enterococcus faecium* during Bologna Sausage Cooking. Lebensm. Wiss. Technol..

[56] Albert I., Mafart P. (2005). A modified Weibull model for bacterial inactivation. Int. J. Food Microbiol..

[57] Baranyi J., Jones A., Walker C., Kaloti A., Robinson T.P., Mackey B.M. (1996). A combined model for growth and subsequent thermal inactivation of *Brochothrix thermosphacta*. Appl. Environ. Microbiol..

[58] Casolari A., Bazin M.J., Prosser J.I. (2009). Microbial death. Physiological Models in Microbiology 2.

[59] Chiruta J., Davey K.R., Thomas C.J., Jowitt R. (1997). Combined effect of temperature and pH on microbial death in continuous pasteurisation of liquids. Engineering and Food at ICEF7.

[60] Daugthry B.J., Davey K.R., Thomas C.J., Verbyla A.P., Jowitt R. (1997). Food processing–A new model for the thermal destruction of contaminating bacteria. Engineering and Food at ICEF7.

[61] Geeraerd A.H., Herremans C.H., Van Impe J.F. (2000). Structural model requirements to describe microbial inactivation during a mild heat treatment. Int. J. Food Microbiol..

[62] Sapru V., Teixeira A.A., Smerage G.H., Lindsay J.A. (1992). Predicting thermophilic spore population dynamics for UHT sterilization processes. J. Food Sci..

[63] Whiting R.C. (1993). Modeling bacterial survival in unfavorable environments. J. Ind. Micro.

[64] Xiong R., Xie G., Edmondson A.E., Sheard M.A. (1999). A mathematical model for bacterial inactivation. Int. J. Food Microbiol..

[65] Aggelis G., Samelis J., Metaxopoulos J. (1998). A novel modelling approach for predicting microbial growth in a raw cured meat product stored at 3 °C and at 12 °C in air. Int. J. Food Microbiol..

[66] Baker D.A., Genigeorgis C. (1990). Predicting the safe storage of fresh fish under modified atmospheres with respect to *Clostridium botulinum* toxigenesis by modeling length of the lag phase of growth. J. Food Prot..

[67] Cauchie E., Delhalle L., Baré G., Tahiri A., Taminiau B., Korsak N., Burteau S., Fall P.A., Farnir F., Daube G. (2020). Modeling the growth and interaction between *Brochothrix thermosphacta*, *Pseudomonas* spp., and *Leuconostoc gelidum* in minced pork samples. Front. Microbiol..

[68] Choi W.-S., Son N., Cho J.-I., Joo I.-S., Han J.-A., Kwak H.-S., Hong J.-H., Suh S.H. (2019). Predictive model of *Staphylococcus aureus* growth on egg products. Food Sci. Biotechnol..

[69] Chung K.-H., Park M.S., Kim H.-Y., Bahk G.J. (2019). Growth prediction and time–temperature criteria model of *Vibrio parahaemolyticus* on traditional Korean raw crab marinated in soy sauce (*ganjang-gejang*) at different storage temperatures. Food Control.

[70] Dickson J.S., Siragusa G.R., Wray J.E. (1992). Predicting the growth of *Salmonella* typhimurium on beef by using the temperature function integration technique. Appl. Environ. Microbiol..

[71] Giannuzzi L., Pinotti A., Zaritzky N. (1998). Mathematical modelling of microbial growth in packaged refrigerated beef stored at different temperatures. Int. J. Food Microbiol..

[72] Hwang C.-A., Huang L. (2019). Growth and survival of *Bacillus cereus* from spores in cooked rice–One-step dynamic analysis and predictive modelling. Food Control.

[73] Hyun J.-E., Yoon J.-H., Lee S.-Y. (2018). Response surface modeling for the inactivation of *Bacillus cereus* on cooked spinach by natural antimicrobial at various temperatures. J. Food Saf..

[74] Juneja V.K., Mishra A., Pradhan A.K. (2018). Dynamic predictive model for growth of *Bacillus cereus* from spores in cooked beans. J. Food Prot..

[75] Juneja V.K., Golden C.E., Mishra A., Harrison M.A., Mohr T.B. (2019). Predictive model for growth of *Bacillus cereus* at temperature applicable to cooling of cooked pasta. J. Food Sci..

[76] Kowalik J., Lobacz A., Zulewska J., Dec B. (2018). Analysis and mathematical modelling of the behaviour of *Escherichia coli* in the mascarpone cheese during cold storage. Int. J. Food Sci. Technol..

[77] Li M., Huang L., Zhu Y., Wei Q. (2019). Growth of *Clostridium perfringens* in roasted chicken and braised beef during cooling–One-step dynamics analysis and modelling. Food Control.

[78] Longhi D.A., da Silva N.B., Martins W.F., Carciofi B.A.M., de Aragão G.M.F., Laurindo J.B. (2018). Optimal experimental design to model spoilage bacteria growth in vacuum-packaged ham. J. Food Eng..

[79] Oscar T.P. (1999). Response surface models for effects of temperature and previous growth sodium chloride on growth kinetics of *Salmonella* typhimurium on cooked chicken breast. J. Food Prot..

[80] Park S.Y., Ha S.-D. (2018). Predictive growth model of the effects of temperature on the growth kinetics of generic *Escherichia coli* in the Korean traditional rice cake product “Garaetteok”. J. Food Sci. Technol..

[81] Park S.Y., Choi S.-Y., Ha S.-D. (2019). Predictive modeling for the growth of *Aeromonas hydrophila* on lettuce as a function of combined storage temperature and relative humidty. Foodborne Pathog. Dis..

[82] Roberts T.A., Gibson A.M., Robinson A. (1981). Prediction of toxin production by *Clostridium botulinum* in pasteurized pork slurry. J. Food Technol..

[83] Taoukis P.S., Koutsoumanis K., Nychas G.J.E. (1999). Use of time-temperature integrators and predictive modelling for shelf life control of chilled fish under dynamic storage conditions. Int. J. Food Microbiol..

[84] Tarlak F., Johannessen G., Villegas I.B., Bolívar A., Posada-Izquierdo G.D., Pérez-Rodríguez F. (2020). Modelling of the behaviour of *Salmonella enterica serovar* Reading on commercial fresh-cut iceberg lettuce stored at different temperatures. Foods.

[85] Vankerschaver K., Willocx F., Smout C., Hendrickx M., Tobback P. (1996). The influence of temperature and gas mixtures on the growth of the intrinsic micro-organisms on cut endive: Predictive versus actual growth. Food Microbiol..

[86] Zamora M.C., Zaritzky N.E. (1985). Modeling of microbial growth in refrigerated packaged beef. J. Food Sci..

[87] Costa J.C.C.P., Bover-Cid S., Bolívar A., Zurera G., Pérez-Rodríguez F. (2019). Modelling the interaction of the sakacin-producing *Lactobacillus sakei* CTC494 and *Listeria monocytogenes* in filleted gilthead sea bream (*Sparus aurata*) under modified atmosphere packaging at isothermal and non-isothermal conditions. Int. J. Food Microbiol..

[88] Cuggino S.G., Bascón-Villegas I., Rincón F., Pérez M.A., Posada-Izquierdo G., Marugán J., Carro C.P., Pérez-Rodríguez F. (2020). Modelling the combined effect of choline, benzyl isothiocyanate, exposure time and cut size on the reduction of *Salmonella* in fresh-cut lettuce during washing process. Food Microbiol..

[89] Jaczynski J., Park J.W. (2003). Predictive models for microbial inactivation and texture degradation in surimi seafood during thermal processing. J. Food Sci..

[90] Jia Z., Li C., Fang T., Chen J. (2019). Predictive modeling of the effect of *ε*-polylysine hydrochloride on growth and thermal inactivation of *Listeria monocytogenes* in fish balls. J. Food Sci..

[91] Juneja V.K., Gonzales-Barron U., Butler F., Yadav A.S., Friedman M. (2013). Predictive thermal inactivation model for the combined effect of temperature, cinnamaldehyde and carvacrol on starvation-stressed multiple *Salmonella* serotypes in ground chicken. Int. J. Food Microbiol..

[92] Juneja V.K., Garcia-Dávila J., Lopez-Romero J.C., Pena-Ramos E.A., Camou J.P., Valenzuela-Melendres M. (2014). Modeling the effects of temperature, sodium chloride, and green tea and their interactions on the thermal inactivation of *Listeria monocytogenes* in Turkey. J. Food Prot..

[93] Koutsoumanis K., Lambropoulou K., Nychas G.-J.E. (1999). A predictive model for the non-thermal inactivation of *Salmonella enteritidis* in a food model system supplemented with a natural antimicrobial. Int. J. Food Microbiol..

[94] Nazarowec-White M., McKellar R.C., Piyasena P. (1999). Predictive modelling of *Enterobacter sakazakii* inactivation in bovine milk during high-temperature short-time pasteurization. Food Res. Int..

[95] Portela J.B., Coimbra P.T., Cappato L.P., Alvarenga V.O., Oliveira R.B.A., Pereira K.S., Azeredo D.R.P., Sant’Ana A.S., Nascimento J.S., Cruz A.G. (2019). Predictive model for inactivation of *Salmonella* in infant formula during microwave heating processing. Food Control.

[96] Santillana Farakos S.M., Pouillot R., Anderson N., Johnson R., Son I., Van Doren J. (2016). Modeling the survival kinetics of *Salmonella* in tree nuts for use in risk assessment. Int. J. Food Microbiol..

[97] Sheen S., Huang C.-Y., Ramos R., Chien S.-Y., Scullen J., Sommers C. (2018). Lethality prediction for *Escherichia Coli* O157:H7 and uropathogenic *E. coli* in ground chicken treated with high pressure processing and trans-cinnamaldehyde. J. Food Sci..

[98] Noriega E., Laca A., Díaz M. (2008). Modelling of diffusion-limited growth to predict *Listeria* distribution in structured model foods. J. Food Eng..

[99] Broughall J.M., Anslow P.A., Kilsby D.C. (1983). Hazard analysis applied to microbial growth in foods: Development of mathematical models describing the effect of water activity. J. Appl. Bacteriol..

[100] Devlieghere F., Geeraerd A.H., Versyck K.J., Vandewaetere J., Van Impe J., Debevere J. (2001). Growth of *Listeria monocytogenes* in modified atmosphere packed cooked meat products: A predictive model. Food Microbiol..

[101] Gibson A.M., Bratchell N., Roberts T.A. (1988). Predicting microbial growth: Growth responses of *salmonellae* in a laboratory medium as affected by pH, sodium chloride and storage temperature. Int. J. Food Microbiol..

[102] Juneja V.K., Marmer B.S., Phillips J.G., Miller A.J. (1995). Influence of the intrinsic properties of food on thermal inactivation of spores of nonproteolytic *Clostridium botulinum*: Development of a predictive model. J. Food Saf..

[103] Aspridou Z., Moschakis T., Biliaderis C.G., Koutsoumanis K.P. (2014). Effect of the substrate’s microstructure on the growth of *Listeria monocytogenes*. Food Res. Int..

[104] Boons K., Van Derlinden E., Mertens L., Peeters V., Van Impe J.F. (2013). Effect of immobilization and salt concentration on the growth dynamics of *Escherichia coli* K12 and *Salmonella* typhimurium. J. Food Sci..

[105] Costello K.M., Gutierrez-Merino J., Bussemaker M., Ramaioli M., Baka M., Van Impe J.F., Velliou E.G. (2018). Modelling the microbial dynamics and antimicrobial resistance development of *Listeria* in viscoelastic food model systems of various structural complexities. Int. J. Food Microbiol..

[106] Kabanova N., Stulova I., Vilu R. (2012). Microcalorimetric study of the growth of bacterial colonies of *Lactococcus lactis* IL1403 in agar gels. Food Microbiol..

[107] Prachaiyo P., McLandsborough L.A. (2003). Oil-in-water emulsion as a model system to study the growth of *Escherichia coli* O157:H7 in a heterogeneous food system. J. Food Sci..

[108] Zalazar A.L., Gliemmo M.F., Campos C.A. (2016). Effect of stabilizers, oil level and structure on the growth of *Zygosaccharomyces bailii* and on physical stability of model systems simulating acid sauces. Food Res. Int..

[109] Castro M.P., Rojas A.M., Campos C.A., Gerschenson L.N. (2009). Effect of preservatives, tween 20, oil content and emulsion structure on the survival of *Lactobacillus fructivorans* in model salad dressings. LWT Food Sci. Technol..

[110] Verheyen D., Baka M., Akkermans S., Skåra T., Van Impe J.F. (2019). Effect of microstructure and initial cell conditions on thermal inactivation kinetics and sublethal injury of *Listeria monocytogenes* in fish-based food model systems. Food Microbiol..

[111] Verheyen D., Govaert M., Seow T.K., Ruvina J., Mukherjee V., Baka M., Skåra T., Van Impe J.F. (2020). The complex effect of food matrix fat content on thermal inactivation of *Listeria monocytogenes*: Case study in emulsion and gelled emulsion model systems. Front. Microbiol..

[112] Bellara S.R., Fryer P.J., McFarlane C.M., Thomas C.R., Hocking P.M., Mackey B.M. (1999). Visualization and modelling of the thermal inactivation of bacteria in a model food. Appl. Environ. Microbiol..

[113] Hamoud-Agha M.M., Curet S., Simonin H., Boillereaux L. (2013). Microwave inactivation of *Escherichia coli* K12 CIP 54.117 in a gel medium: Experimental and numerical study. J. Food Eng..

[114] Mackey B.M., Kelly A.F., Colvin J.A., Robbins P.T., Fryer P.J. (2006). Predicting the thermal inactivation of bacteria in a solid matrix: Simulation studies on the relative effects of microbial thermal resistance parameters and process conditions. Int. J. Food Microbiol..

[115] Noriega E., Laca A., Díaz M. (2008). Modelling of diffusion-limited growth for food safety in simulated cheeses. Food Bioprod. Process..

[116] Mertens L., Van Derlinden E., Dang T.D.T., Cappuyns A.M., Vermeulen A., Debevere J., Moldenaers P., Devlieghere F., Geeraerd A.H., Van Impe J.F. (2011). On the critical evaluation of growth/no growth assessment of *Zygosaccharomyces bailii* with optical density measurements: Liquid versus structured media. Food Microbiol..

[117] Ter Steeg P.F., Otten G.D., Alderliesten M., De Weijer R., Naaktgeboren G., Bijl J., Vasbinder A.J., Kershof I., Van Duijvendijk A.M. (2001). Modelling the effects of (green) antifungals, droplet size distribution and temperature on mould outgrowth in water-in-oil emulsions. Int. J. Food Microbiol..

[118] Kapetanakou A.E., Ampavi A., Yanniotis S., Drosinos E.H., Skandamis P.N. (2011). Development of a model describing the effect of temperature, water activity and (gel) structure on growth and ochratoxin A production by *Aspergillus carbonarius* in vitro and evaluation in food matrices of different viscosity. Food Microbiol..

[119] Dens E.J., Van Impe J.F. (2001). On the need for another type of predictive model in structured foods. Int. J. Food Microbiol..

[120] De Bonis M.V., Ruocco G. (2016). A heat and mass transfer perspective of microbial behavior modelling in a structured vegetable food. Int. J. Food Eng..

[121] Ferrier R., Hezard B., Lintz A., Stahl V., Augustin J.-C. (2013). Combining individual-based modelling and food microenvironment descriptions to predict the growth of *Listeria monocytogenes* on smear soft cheese. Appl. Environ. Microbiol..

[122] Augustin J.-C., Ferrier R., Hezard B., Lintz A., Stahl V. (2015). Comparison of individual-based modeling and population approaches for prediction of foodborne pathogens growth. Food Microbiol..

[123] Vereecken K.M., Devlieghere F., Bockstaele A., Debevere J., Van Impe J.F. (2003). A model for lactic acid-induced inhibition of *Yersinia enterocolitica* in mono- and coculture with *Lactobacillus sakei*. Food Microbiol..

[124] Ross T., Ratkowsky D.A., Mellefont L.A., McMeekin T.A. (2003). Modelling the effects of temperature, water activity, pH and lactic acid concentration on the growth rate of *Escherichia coli*. Int. J. Food Microbiol..

[125] Reiner M. (1926). Über die strömung einer elastichen flüssigkeit durch eine kapillare. Kolloid, Z..

[126] Verheyen D., Bolívar A., Pérez-Rodríguez F., Baka M., Skåra T., Van Impe J.F. (2020). Isolating the effect of fat content on *Listeria monocytogenes* growth dynamics in fish-based emulsion and gelled emulsion systems. Food Control.

[127] Van Impe J.F., Poschet F., Geeraerd A.H., Vereecken K.M. (2005). Towards a novel class of predictive microbial growth models. Int. J. Food Microbiol..

[128] Tack I. (2016). Metabolic Differentiation in Microbial Colonies and Biofilms: A Multiscale Modelling Approach. Ph.D. Thesis.

[129] Ayala F.J., Gilpin M.J., Ehrenfield J.G. (1973). Competition between species: Theoretical models and experimental results. Theor. Pop Biol..

[130] Bailey J.E., Ollis D.F. (1986). Biochemical Engineering Fundamentals.

[131] Baranyi J., Robinson T.P., Kaloti A., Mackey B.M. (1995). Predicting the growth of *Brochothrix thermosphacta* at changing temperature. Int. J. Food Microbiol..

[132] Ratkowsky D.A., Olley J., McMeekin T.A., Ball A. (1982). Relationship between temperature and growth rate of bacterial cultures. J. Bacteriol..

[133] Gorochowski T.E., Matyjaszkiewicz A., Todd T., Oak N., Kowalska K., Reid S., Tsaneva-Atanasova K.T., Savery N.J., Grierson C.S., di Bernardo M. (2012). BSim: An agent-based tool for modeling bacterial populations in systems and synthetic biology. PLoS ONE.

[134] González-Cabaleiro R., Mitchell A.M., Smith W., Wipat A., Ofiteru I.D. (2017). Heterogeneity in pure microbial systems: Experimental measurements and modeling. Front. Microbiol..

[135] Kreft J.-U., Booth G., Wimpenny J.W.T. (1998). BacSim, a simulator for individual-based modelling of bacterial colony growth. Microbiology.

[136] Ginovart M., López D., Valls J. (2002). INDISIM, an individual-based discrete simulation model to study bacterial cultures. J. Theor. Biol..

[137] Verhulst A.J., Cappuyns A.M., Van Derlinden E., Bernaerts K., Van Impe J.F. (2011). Analysis of the lag phase to exponential growth transition by incorporating inoculum characteristics. Food Microbiol..

[138] Tack I.L.M.M., Nimmegeers P., Akkermans S., Hashem I., Van Impe J.F.M. (2017). Simulation of *Escherichia coli* dynamics in biofilms and submerged colonies with an individual-based model including metabolic network information. Front. Microbiol..

[139] Lardon L.A., Merkey B.V., Martins S., Dötsch A., Picioreanu C., Kreft J.-U., Smets B.F. (2011). iDynoMiCS: Next-generation individual-based modelling of biofilms. Environ. Microbiol..

[140] Hellweger F.L., Clegg R.J., Clark J.R., Plugge C.M., Kreft J.U. (2016). Advancing microbial sciences by individual-based modelling. Nat. Rev. Microbiol..

[141] Possas A., Pérez-Rodríguez F., Valero A., Rincón F., García-Gimeno R.M. (2018). Mathematical approach for the *Listeria monocytogenes* inactivation during high hydrostatic pressure processing of a simulated meat medium. Innov. Food Sci. Emerg. Technol..

[142] Chhabra A.T., Carter W.H., Linton R.H., Cousin M.A. (1999). A predictive model to determine the effects of pH, milkfat, and temperature on thermal inactivation of *Listeria monocytogenes*. J. Food Prot..

[143] Santillana Farakos S.M., Frank J.F., Schaffner D.W. (2013). Modeling the influence of temperature, water activity and water mobility on the persistence of *Salmonella* in low-moisture foods. Int. J. Food Microbiol..

[144] Trimble L.M., Frank J.F., Schaffner D.W. (2020). Modification of a predictive model to include the influence of fat content on *Salmonella* inactivation in low-water-activity foods. J. Food Prot..

[145] Albuquerque C.D.D., Curet S., Boillereaux L. (2019). A 3D-CFD-heat-transfer-based model for the microbial inactivation of pasteurized food products. Innov. Food Sci. Emerg. Technol..

[146] Gil M.M., Miller F.A., Brandão T.R.S., Silva C.L.M. (2011). On the use of the Gompertz model to predict microbial thermal inactivation under isothermal and non-isothermal conditions. Food Eng. Rev..

[147] Mafart P., Couvert O., Gaillard S., Leguerinel I. (2002). On calculating sterility in thermal preservation methods: Application of the Weibull frequency distribution model. Int. J. Food Microbiol..

[148] Huang L. (2009). Thermal inactivation of *Listeria monocytogenes* in ground beef under isothermal and dynamic temperature conditions. J. Food Eng..

[149] Chen H.-H., Kang H.-Y., Chen S.-D. (2008). The effects of ingredients and water content on the rheological properties of batters and physical properties of crusts in fried foods. J. Food Eng..

[150] Rao M.A. (2007). Rheology of Fluid and Semifluid Foods: Principles and Applications.

[151] Whiting R.C., Sackitey S., Calderone S., Morely K., Phillips J.G. (1996). Model for the survival of *Staphylococcus aureus* in nongrowth environments. Int. J. Food Microbiol..

[152] Bhuvaneswari E., Anandharamakrishnan C. (2014). Heat transfer analysis of pasteurization of bottled beer in a tunnel pasteurizer using computational fluid dynamics. Innov. Food Sci. Emerg. Technol..

[153] Erdogdu F., Tutar M., Sarghini F., Skipnes D. (2017). Effects of viscosity and agitation rate on temperature and flow field in cans during reciprocal agitation. J. Food Eng..

[154] Topcam H., Karatas O., Erol B., Erdogdu F. (2020). Effect of rotation on temperature uniformity of microwave processed low-high viscosity liquids: A computational study with experimental validation. Innov. Food Sci. Emerg. Technol..

[155] Wu H., Karayiannis T.G., Tassou S.A. (2013). A two-dimensional frying model for the investigation and optimisation of continuous industrial frying systems. Appl. Therm. Eng..

[156] Bedane T.F., Erdogdu F., Lyng J.G., Marra F. (2021). Effects of geometry and orientation of food products on heating uniformity during radio frequency heating. Food Bioprod. Process..

[157] Erdogdu F., Karatas O., Sarghini F. (2018). A short update on heat transfer modelling for computational food processing in conventional and innovative processing. Curr. Opin. Food Sci..

[158] Aspridou A., Koutsoumanis K.P. (2015). Individual cell heterogeneity as variability source in population dynamics of microbial inactivation. Food Microbiol..

[159] Abe H., Koyama K., Kawamura S., Koseki S. (2018). Stochastic evaluation of *Salmonella enterica* lethality during thermal inactivation. Int. J. Food Microbiol..

[160] Koseki S., Koyama K., Abe H. (2021). Recent advances in predictive microbiology: Theory and application of conversion from population dynamics to individual cell heterogeneity during inactivation process. Curr. Opin. Food Sci..

